# OCT-Based Analysis of Post-Lens Tear Film Stability in Silicone Hydrogel Contact Lenses

**DOI:** 10.22336/rjo.2025.59

**Published:** 2025

**Authors:** Neşe Arslan, Şule Barman Kakil

**Affiliations:** 1 Department of Ophthalmology, Dışkapi Yıldırım Beyazıt Training and Research Hospital, Ankara, Turkey; 2 Department of Ophthalmology, Tayfur Ata Sökmen Faculty of Medicine, Hatay Mustafa Kemal University, Hatay, Turkey

**Keywords:** contact lens, optical coherence tomography, post-lens tear film thickness, tear film stability, CL = Contact Lens, CLIDE = Contact Lens-Induced Dry Eye, NIAvgBUT = Non-Invasive Average Break-Up Time, OCT = Optical Coherence Tomography, PLTF = Pre-Lens Tear Film, PoLTF = Post-Lens Tear Film, SD = Standard Deviation, SPSS = Statistical Package for the Social Sciences

## Abstract

**Purpose:**

This study aimed to evaluate the stability of post-lens tear film (PoLTF) and pre-lens tear film (PLTF) in three different silicone hydrogel contact lenses (CLs) using optical coherence tomography (OCT) and multifunctional topography.

**Materials and methods:**

A total of 158 participants were assigned to three groups based on the CL material: Group 1 (Lotrafilcon B), Group 2 (Senofilcon A), and Group 3 (Samfilcon A). Non-invasive average break-up time (NIAvgBUT) of the PLTF was measured after a few hours of lens wear during the first visit and after 2-4 weeks of continued use in the second visit. PoLTF thickness was assessed in five corneal quadrants using OCT, measured both indirectly and manually.

**Results:**

The mean participant age was 21.9±5.0 years. The NIAvgBUT of the PLTF was significantly higher at the first visit than at the second visit (9.5±2.9 s vs. 8.3±2.1 s, p=0.0001). Similarly, the PoLTF thickness in the central corneal area showed a significant reduction after 2-4 weeks of lens wear (p=0.001).

**Discussion:**

Our results emphasize the clinical importance of monitoring both pre-lens and post-lens tear film parameters during routine follow-up visits. By integrating OCT-based measurements into daily practice, clinicians may identify early tear film instability and prevent contact lens intolerance. This is particularly relevant for younger patients who are at risk of long-term ocular surface changes due to extended lens wear.

**Conclusion:**

A significant decrease in PLTF stability (NIAvgBUT) and PoLTF thickness in the central corneal area was observed after 2-4 weeks of CL use. These findings suggest that prolonged CL wear affects tear film stability and surface wettability, highlighting the importance of monitoring these changes over time.

## Introduction

Globally, more than 140 million people wear contact lenses (CLs), yet approximately 50% report experiencing dry eye symptoms, leading to discontinuation rates ranging from 12% to 51% [1,2]. A stable tear film maintains ocular surface health, enables smooth blinking, and minimizes CL-related friction [3-5].

When a CL is placed on the eye, the tear film is divided into the pre-lens tear film (PLTF) and post-lens tear film (PoLTF), altering its structure and stability. This separation modifies tear film composition, disrupts its protective function, and increases tear evaporation, which may contribute to contact lens-induced dry eye (CLIDE) [4-6].

The PoLTF plays a crucial role in cushioning the CL against the cornea, maintaining comfort and visual clarity. At the same time, the PLTF, particularly its lipid layer, provides a uniform optical surface and maintains contact lens wettability and prevents excessive tear evaporation [6-9]. Disruptions in either layer compromise lens performance, reduce wearing comfort, and increase the risk of ocular surface damage. Given that CLIDE remains a leading cause of contact lens intolerance, assessing both PLTF and PoLTF parameters can provide valuable insights into optimizing contact lens materials and improving user comfort over extended wear periods [10-14].

While several methods exist to measure tear film thickness, recent studies have demonstrated that optical coherence tomography (OCT) offers a non-invasive, high-resolution approach to evaluating tear film stability and lens-surface interactions [14,15].

In this study, we aimed to indirectly measure PoLTF thickness using the pachymetry mode of anterior segment OCT and assess non-invasive tear break-up time (NIAvgBUT) over the CL surface in users of various new-generation silicone hydrogel (SiHy) CLs. By integrating OCT-based measurements, we aimed to provide valuable insights into tear film stability, thereby aiding in the development of improved CL designs and enhancing the comfort and longevity of CL wear.

## Materials and methods

This study adhered to the principles outlined in the Declaration of Helsinki for human research and received approval from the Ethics Committee of Diskapi Yildirim Beyazit Education and Research Hospital, University of Health Sciences.

A total of 158 participants presenting to our clinic for spherical contact lens (CL) fitting were enrolled. Exclusion criteria included a history of CL use, smoking, clinically diagnosed dry eye disease, any ocular or systemic pathology, and medication use known to cause ocular side effects. Pregnant or lactating individuals were also excluded. Participants were selected to ensure an even distribution across the specified age range (16-41 years). Each study group contained individuals spanning this range to maintain a balanced representation across different age groups. The mean ages of the groups were statistically analyzed to confirm comparability.

Participants were categorized into three groups based on the trial silicone hydrogel (SiHy) CL type: Group 1: Lotrafilcon B, Group 2: Senofilcon A, and Group 3: Samfilcon A.

### Examination Protocol

All measurements were conducted on the right eye of each participant to standardize the measurements and avoid inter-eye variability. To minimize the potential influence of overnight corneal edema, examinations were scheduled after 10 AM. All procedures were performed in a dimly lit environment by the same experienced ophthalmologist.

All contact lenses were used strictly within their manufacturer-recommended replacement schedules: Senofilcon A: 2 weeks, Lotrafilcon B: 4 weeks, Samfilcon A: 4 weeks. Participants who extended lens use beyond the prescribed period were excluded from the study.

**Fig. 1:**
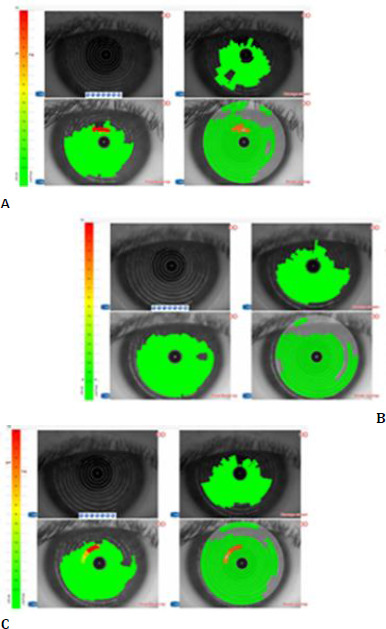
Non-invasive break-up time (NIAvgBUT) of the PLTF. **A**. NIAvgBUT of the cornea. **B**. NIAvgBUT of the PLTF in the first visit after 2 hours of CL application. **C**. NIAvgBUT of the PLTF after 2-4 weeks of using the same CL

Non-invasive tear break-up time (NIAvgBUT) was assessed using a Sirius Scheimpflug Camera (Bon Optic VertriebsgmbH, Lübeck, Germany). The NIAvgBUT was evaluated 1-2 hours after CL application during the first visit and after 2-4 weeks of continuous wear during the follow-up visit (**Fig. 1**).

Since Lotrafilcon B and Samfilcon A feature surface treatment properties, an additional comparison was made between treated and untreated CL groups regarding tear film stability [12].

**Fig. 2:**
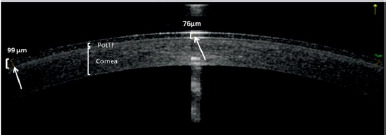
Measurement of PoLTF based on the space between the CL and the cornea using the pachymetry mode of the OCT

PoLTF was measured indirectly using anterior segment OCT (Optovue RTVue-XR, Optovue Inc., USA) in pachymetry mode (**Fig. 2**). The tear film thickness between the posterior CL surface and the cornea was assessed at five quadrants: central, superior, inferior, temporal, and nasal. Measurements were performed over a 6 × 6 mm grid area. Each measurement was repeated three times, and the mean value was recorded for further analysis. Corneal thickness with the trial CL was recorded at the first and follow-up visits (2 weeks for Senofilcon A, 4 weeks for Lotrafilcon B, and Samfilcon A). All contact lenses used in this study were within a power range of -0.50 D to -6.00 D. Since contact lens power is directly related to lens thickness, variations in lens power could potentially influence PoLTF thickness measurements.

To minimize this effect, only spherical lenses with similar base curves and diameters were selected. Additionally, measurements were consistently taken at the central corneal area, where lens thickness variation is minimal across different dioptric powers.

Tear film stability parameters and PoLTF thickness variations were compared among the three CL groups. The impact of CL material and surface treatment on tear film stability was further analyzed.

### Statistical analysis

All analyses were performed using IBM SPSS Statistics 20 (IBM Corp., Armonk, NY, USA). Data normality was assessed with the Shapiro-Wilk test. Continuous variables were reported as mean ± standard deviation (SD), and categorical data as frequency (%).

For normally distributed variables, ANOVA with Bonferroni correction was used, while Kruskal-Wallis and Dunn’s tests were applied for non-normally distributed data. Intra-group changes in NIAvgBUT and PoLTF thickness were analyzed using paired t-tests for normal data and Wilcoxon signed-rank tests for non-parametric data. Chi-square or Fisher’s exact test was used for categorical comparisons.

Pearson’s correlation was applied for normally distributed relationships, while Spearman’s correlation was used otherwise. Effect sizes were reported with Cohen’s d (paired t-tests) and eta-squared (ANOVA). Odds ratios (ORs) and 95% confidence intervals (CIs) were calculated for categorical associations. A P-value of <0.05 was considered statistically significant.

## Results

A total of 158 right eyes from 118 females (74.7%) and 40 males (25.3%) were included in the study. The mean age of the participants was 21.9±5.0 years (range: 16-41 years). The participants’ age range was 16-41 years, with all study groups including individuals across this spectrum. There were no significant differences in mean age among the groups (P=0.052), ensuring that age distribution did not introduce a bias in the results.

The distribution of participants across the study groups was as follows: Group 1 (Lotrafilcon B, n=54), Group 2 (Senofilcon A, n=54), and Group 3 (Samfilcon A, n=50). There were no statistically significant differences between the groups in terms of age and sex (P>0.05).

The mean NIAvgBUT values of the pre-lens tear film (PLTF) were 9.5±2.9 seconds at the first visit and 8.3±2.1 seconds at the second visit. A significant reduction in NIAvgBUT was observed between the first and second visits (P=0.0001). However, there was no statistically significant difference in NIAvgBUT values among the study groups (P>0.05, **Table 1**). When comparing the surface-treated CLs (Lotrafilcon B, Samfilcon A) with the non-treated CL (Senofilcon A), no significant differences were found in NIAvgBUT values between the first and second visits (P=0.9).

**Table 1:** The NIAvgBUTs on the CL surfaces during the first and second visits

NIAvgBUT *(second)*	Lotrafilcon B	Senofilcon A	Samfilcon A	p
**Lens surface in the 1^st^ visit**	9.96±3.2	9.2±2.8	9.4±2.6	0.35
**Lens surface in the 2^nd^ visit**	8.5±2.6	7.9±1.6	8.4±1.9	0.25

NIAvgBUT = non-invasive tear breakup time average

The mean PoLTF thickness values for each corneal quadrant during the first and second visits are presented in **Table 2**. At the same time, there were no significant differences in PoLTF thickness among the study groups (P>0.05). A statistically significant reduction in PoLTF thickness was observed in the central area between the first and second visits (P=0.001). Specifically, PoLTF thickness in the central area was significantly lower at the second visit in Group 1 (Lotrafilcon B, P=0.0001) and Group 3 (Samfilcon A, P=0.001).

**Table 2:** Mean PoLTF thickness measurements for all the quadrants during the first and second visits

Area	PoLTF µm Mean±SD	p value
**Central area 1^st^** **Central area 2^nd^**	53.2±2.0 52.4±2.5	0.001*
**Superior quadrant 1^st^** **Superior quadrant 2^nd^**	86.8±7.7 86.6±7.4	0.68
**Inferior quadrant 1^st^** **Inferior quadrant 2^nd^**	85.3±9.3 85.3±9.1	0.95
**Nasal quadrant 1^st^** **Nasal quadrant 2^nd^**	86.6±8.5 87.4±8.2	0.18
**Temporal quadrant 1^st^** **Temporal quadrant 2^nd^**	83.9±9.2 84.4±9.3	0.43

PoLTF = Post-Lens tear film thickness* Bold text indicates statistical significance

## Discussion

Soft contact lenses (CLs) vary in material composition and surface properties, with their stability influenced by factors such as replacement frequency, temperature, and time. These variations affect tear film stability and ocular surface integrity [16]. The pre-lens tear film (PLTF) and post-lens tear film (PoLTF) play a crucial role in maintaining comfort and optimal vision. This study utilized optical coherence tomography (OCT) and multifunctional topography to evaluate the stability of PoLTF and PLTF in three different silicone hydrogel (SiHy) CLs [3].

Silicone hydrogel CLs are designed with surface treatment technologies, such as plasma coating and moisture retention systems, to enhance wettability and overall performance [14]. Maintaining optimal surface wettability is essential for preventing lens-related dryness and ensuring long-term comfort during wear. While some studies have shown variations in wettability among different lens materials, others have found no statistically significant differences. However, the long-term impact of lens wear on tear film stability remains a key concern [6,15].

PLTF stability is essential for lens performance. While previous studies reported no significant differences in tear break-up time (TBUT) among different lenses, our findings highlight the importance of assessing PLTF changes over time [3]. Using a non-invasive break-up time (NIAvgBUT) measurement technique, we identified a significant reduction after 2-4 weeks of continuous lens use. This decline suggests that SiHy lenses experience progressive changes in surface wettability and oxygen permeability over time.

The decline in NIAvgBUT suggests that SiHy lenses progressively lose surface wettability and oxygen permeability as they approach the end of their recommended replacement period. Previous studies have demonstrated that wettability decreases with lens aging and surface alterations [17], and that long-term disposable lens use carries both benefits and risks in terms of ocular surface health [18]. Moreover, poor compliance with replacement schedules has been associated with reduced tear film stability and an increased risk of contact lens-related complications [19,20]. Together, these findings support the view that inadequate lens replacement contributes significantly to the development of CL-induced dry eye. Adhering to the manufacturer’s recommended replacement schedules is essential to prevent discomfort and complications. However, many CL users do not follow proper lens care and replacement guidelines, with delayed replacement being a common issue [21-23]. Given the importance of lens surface integrity in maintaining tear film stability, patient education and compliance with replacement schedules are crucial to reducing dryness and irritation.

Dumbleton et al. [23] reported a link between contact lens replacement compliance and the risk of lens-related complications. As tear film stability declines near the end of a lens’s lifespan, prolonged use increases discomfort, dryness, and ocular surface disturbances. Since CLs undergo structural and material changes over time, monitoring post-lens tear film (PoLTF) stability can help assess lens aging effects.

Our findings suggest that PoLTF and pre-lens tear film (PLTF) measurements could be valuable in clinical practice for evaluating lens performance and ocular surface health. The PoLTF assessment provides objective data on tear film dynamics, potentially allowing for the early detection of contact lens-induced dry eye before symptoms appear. Additionally, PLTF stability measurements may help determine the most suitable lens material for patients with pre-existing tear film instability. These findings support the need for OCT-based PoLTF assessment to improve CL fitting and minimize lens-related discomfort.

Integrating these assessments into routine lens evaluations may enhance personalized lens selection, improve comfort, and reduce the risk of long-term ocular complications. Future research should explore their predictive value in identifying individuals at risk for contact lens intolerance.

This study demonstrated that PoLTF thickness can be indirectly measured using the pachymetry mode of anterior segment OCT, offering a non-invasive and precise method for tracking post-lens tear film changes. The similar results across all CL groups may be due to the comparable optical design and material properties of Lotrafilcon B, Senofilcon A, and Samfilcon A. Since these lenses share high oxygen permeability, their impact on tear film stability was likely comparable. However, prior studies indicate that differences in material composition, surface coatings, and water content can influence tear film dynamics [24,25]. Future research comparing SiHy lenses with hydrogel lenses or different optical zone designs may provide further insights.

To the best of our knowledge, this study is the first to indirectly measure the space between the cornea and the contact lens (CL) using the pachymetry mode of OCT across five distinct regions (central, superior, nasal, inferior, and temporal) for three different silicone hydrogel (SiHy) CLs.

Previous studies have used various methods to assess pre-corneal and post-lens tear film thickness. Prydal and Campbell [26] and Prydal et al. [27] estimated pre-corneal tear film thickness to be 34-45 µm. Wang et al. [2,8] found that CL insertion significantly increased pre-corneal tear film thickness and indirectly measured PoLTF for two lenses (Night and Day Lotrafilcon A and Senofilcon B), reporting values of 4.5±2.3 µm and 4.7±3.1 µm, respectively. Their formula for PoLTF was PoLTF = C3-C2, where C3 represents corneal thickness with the CL and PoLTF, and C2 includes the PLTF. Lin et al. [6] reported PoLTF thickness between 11 and 12 µm using optical pachymetry.

In our study, PoLTF thickness was measured using the pachymetry mode of anterior segment OCT. To ensure precision, the same ophthalmologist performed all measurements. The mean PoLTF thickness in the central area was 53.2±2.0 µm, which is higher than previously reported values.

This discrepancy may be due to several factors. First, we utilized the pachymetry mode of anterior segment OCT, whereas previous studies employed interferometry, optical pachymetry, or various OCT protocols. Second, sample size variations could be a factor, as our study included 158 participants, whereas prior studies had smaller cohorts (9 and 40 participants, respectively). Third, the specific properties of the SiHy CLs in our study (Lotrafilcon B, Senofilcon A, and Samfilcon A), including surface treatments and material composition, may have influenced PoLTF thickness. Additionally, the duration of CL wear before measurement varied between studies. While some assessed PoLTF shortly after lens insertion, our measurements were taken after 2-4 weeks of continuous wear, allowing for surface changes and tear film adaptation. Lastly, differences in tear film physiology, corneal curvature, tear osmolarity, and ocular surface homeostasis may also explain the variations [11].

### Strengths and Limitations

Unlike previous studies relying on interferometry or optical pachymetry, our method enabled direct visualization and more precise thickness estimation. Measuring PoLTF in multiple quadrants allowed for a comprehensive evaluation of tear film stability, which is essential for optimizing CL comfort and preventing dry eye symptoms. Additionally, our comparison of three SiHy CLs with varying material properties provided insights into how CL design affects tear film behavior over time. The inclusion of follow-up measurements over 2-4 weeks further enhanced our understanding of long-term changes, unlike most prior studies that focused on immediate effects. Another strength is the use of NIAvgBUT via multifunctional topography, providing a clinically relevant tear film stability metric. Our relatively large sample size (n=158) enhanced statistical reliability. Combining OCT-based PoLTF measurements with tear film stability assessments introduces a new framework for evaluating CL performance and optimizing lens fitting strategies.

However, our study had limitations. It did not include hydrogel (Hy) CLs, which could have provided a more comprehensive comparison of material effects on tear film stability. Additionally, best-corrected visual acuity (BCVA) or uncorrected visual acuity (UCVA) was not evaluated, which could provide further insight into how the tear film stability could affect the visual quality. Future research incorporating these factors would offer a broader perspective. Variations in optical zone design among the tested SiHy lenses may have also influenced PoLTF measurements.

## Conclusion

In conclusion, this study highlights the importance of assessing PLTF stability and PoLTF thickness using OCT and multifunctional topography in CL fitting practice. Our findings suggest that prolonged CL wear affects surface wettability and tear film stability, impacting ocular comfort, offering insights into how different SiHy CLs influence tear film dynamics. While PLTF and PoLTF measurements provide valuable data, their routine clinical application is unclear. Further research should explore their role in CL fitting and patient-specific lens selection.

Integrating OCT into clinical practice enables non-invasive, quantitative CL assessments, supporting more personalized and effective fitting strategies.

## Data Availability

The datasets used and analyzed during the current study are available from the corresponding author upon reasonable request.

## Permission to Reproduce Material from Other Sources

Not applicable; no previously published material was reproduced in this study.

## Clinical Trial Registration

Not applicable; this study does not involve a registered clinical trial.
